# *Solanum baumii* (Solanaceae), a New Species From Northeastern Thailand

**DOI:** 10.1155/tswj/9243315

**Published:** 2025-06-18

**Authors:** Wannachai Chatan, Phukphon Munglue, Wilawan Promprom

**Affiliations:** ^1^Department of Biology, Faculty of Science, Mahasarakham University, Maha Sarakham, Thailand; ^2^Plant and Innovation Research Unit, Mahasarakham University, Maha Sarakham, Thailand; ^3^Program of Biology, Faculty of Science, Ubon Ratchathani Rajabhat University, Ubon Ratchathani, Thailand

**Keywords:** Asia, eudicots, plant diversity, Solanales, spiny solanums, taxonomy

## Abstract

*Solanum baumii* Chatan and Promprom, a new species from Mukdahan Province, Phu Pha Yol National Park, northeastern Thailand, is morphologically described and illustrated. It is similar to *Solanum barbisetum* Nees and *Solanum praetermissum* Kerr ex Barnett, but it clearly differs from two latter species in several ways. Its young stems are armed with very few and thin prickles, which become unarmed as they age, with brownish-green prickles in live plants. The flowers are 4–5-merous, and the calyx tube is 5.0–5.5 mm long with brownish-green prickles, while the corolla is always white. The androecium consists of 4–5 stamens. The ovaries are glabrous except for sparsely glandular hairs near the apex, and the fruits are 1.0–1.2 cm in diameter, green at maturity. A key to these closely related species is presented. The preliminary conservation status was assessed, and the distinct morphological characters between the new and similar species were discussed.

## 1. Introduction

*Solanum* L. is a large genus in the family Solanaceae. It is estimated that there are approximately 1240–1300 species in this genus [1–3]. *Solanum* is distributed across all temperate and tropical continents. *Solanum* species have flowers with fused sepals and petals that are usually 5-merous, stellate to pentagonal corollas, stamens with short filaments, and anthers that open by terminal pores. The highest diversity of the genus is in tropical South America, concentrated in a circle around the Amazon basin [1]. This genus includes economically important plants, such as the cultivated potato (*Solanum tuberosum* L.), tomato (*Solanum lycopersicum* L.), and aubergine (*Solanum melongena* L.), and some species are a very rich source of polyphenol compounds with antioxidant properties, such as eggplant (*S. melongena* L.) [4]. About 21 species in this genus are estimated to be found in Thailand [5].

The genus *Solanum* is divided into 12 major clades, with the Leptostemonum Clade being the most species-rich, containing 578 out of the 1244 recognized species [1, 6]. Known traditionally as subgenus *Leptostemonum*, this clade is distinguished by tapered anthers, stellate trichomes, and epidermal prickles and has been recognized as a distinct group since 1753 [7, 8]. Although most of its diversity is in the Americas, the clade also has significant species richness in Australia and tropical Asia.

Molecular phylogenetic studies have established that the spiny solanums from Africa, Asia, and Australia form a monophyletic group, though earlier analyses included few tropical Asian species [9–13] which expanded the focus to include 60 species from Asia and Australia, confirming that most African, Asian, and Australian species belong to a large, monophyletic group known as the Eastern Hemisphere spiny clade, previously called the “Old World clade.” This clade includes well-supported subgroups with mixed geographic origins, though some South American clades (Torva and Lasiocarpum) contain a few species from Asia and the Australo-Pacific region, suggesting long-distance dispersal. While African species form previously known clades, many tropical Asian species cluster in large polytomies with Australian, New Guinean, and Pacific species, with only a few embedded in African clades. The poorly resolved phylogenetic structure limits detailed analysis of biogeography and trait evolution for tropical Asian spiny solanums, and further study is needed, especially including more Pacific, Australian, and New Guinean species where ongoing taxonomic work is expanding species knowledge.

During field trips for collecting plants in the genus *Solanum* in northeastern Thailand from 2018 to 2021, the authors found that this region has high plant diversity and a significant number of medicinal plants. Many local ethnic groups and herbalists use these local plants for their everyday activities. The plant specimens from these areas were collected for study. Among them are *Solanum* specimens belonging to the subgenus *Leptostemonum*, which are slightly similar to previously described *Solanum* species, that is, *Solanum praetermissum* Kerr ex Barnett [1, 14, 15] and *Solanum barbisetum* Nees ([1, 15, 16]. After carefully comparing them, the authors found that the specimens differed from *S. praetermissum* Kerr ex Barnett and *S. barbisetum* Nees. Consequently, a new species is presented here.

## 2. Materials and Methods

The plant specimens were collected from Phu Pha Yol National Park in 2021, and some specimens were planted in the first author's home garden. The morphological characters of the new species were studied on living plants from the field, the author's home garden, and herbarium specimens in BKF. Measurements were made with a vernier caliper or an ocular micrometer in a dissecting microscope.

This study consulted the online databases of type specimens of similar species in K (*S. praetermissum* Kerr ex Barnett (E. Smith *s.n*., holotype) and *S. barbisetum* Nees (N. Gómez 9071, lectotype)) [3] and the JSTOR Global Plants database (http://plants.jstor.org/), the first protologues [14, 16], and the relevant taxonomic literature, such as [1, 17–20]. The preparation of the plant description is based on the terminology presented by [21] and incorporates insights from [1, 2]. The preliminary conservation status of the new species was assessed by applying the criteria given by the IUCN Standards and Petitions Committee [22].

## 3. Taxonomic Treatment

### 3.1. *Solanum baumii* Chatan and Promprom, sp. nov. (Figures 1, 2, and 3)

## 4. Type

Thailand. Mukdahan Province, Phu Pha Yol National Park, northeastern Thailand, 300–400 m elevation, 16°45⁣′04.1⁣^″^N, 104°15⁣′35.5⁣^″^E, 29 August 2021, W. Chatan *3495* (holotype: BKF!; isotype: BK!).

## 5. Diagnosis

*Solanum baumii* Chatan and Promprom is similar to *S. barbisetum* Nees and *S. praetermissum* Kerr ex Barnett, but the new species clearly differs from two similar species in several ways. Its young stems are armed with very few and thin prickles, which become unarmed as they age, with brownish-green prickles in live plants. The flowers are 4–5-merous, and the calyx tube is 5.0–5.5 mm long with brownish-green prickles, while the corolla is always white. The androecium consists of 4–5 stamens. The ovaries are glabrous except for sparsely glandular hairs near the apex, and the fruits are 1.0–1.2 cm in diameter, green at maturity.

## 6. Description

Small shrub, 40–70 m high. Stems armed with very few and thin prickles when young, the older stems unarmed; the prickles erect, 0.5–1.5 mm long, 1.0–1.5 mm diam. at the base, brownish green; moderately to densely pubescent with white stellate trichomes, stellate hairs with the stalks ca. 1 mm long, rays 6–9 and ca. 0.5–1.5 mm long; the older stems becoming sparsely pubescent to nearly glabrous. Leaves simple; blades 9.0–13.5 × 7.0–9.5 cm, ovate or elliptic or circular, mucronate–apiculate or retuse apex, cuneate to truncate and oblique base, sinuate margin, chartaceous, green on both surfaces with the adaxial side typically darker; adaxial surface covered by stellate trichomes with 1–7 rays, the more densely hairs on midrib and veins; armed with a few prickles 1–3 mm long on the lower 2/3 of midrib and base of lateral veins; the abaxial surface more densely stellate trichomes than on the adaxial surface and very dense ones on midrib, lateral vein, and veins, armed with a few prickles 1–3 mm long on the lower half or near the base of midrib or absent, venation pinnate, the secondary veins 3–5 on each side of the midvein; petioles 2.0–5.5 cm, densely pubescent with stellate trichomes like those of the stem, armed with few prickles like those of the stem. Inflorescence extra-axillary scorpioid racemes 3–4 cm long, bearing 6–18 flowers; peduncle 4–6 mm; rachis 3–6 cm; pedicels 9–12 mm long with short prickles, curved, 12–14 mm and 5-ridges in fruit, spaced 1–3 mm apart, articulated at the base. Flowers 4–5-merous, actinomorphic, apparently all perfect; the floral buds ovoid to slightly narrowly ovoid, 7–10 mm long. Calyx tube 5 − 5.5 mm long in the bud through anthesis, abaxial side with brownish-green prickles in living plants, cupular with 4–5 lobes, abaxial surface one medial ridge and densely pubescent with stellate trichomes like those of the stem, adaxial surface glabrescent or with few stellate trichomes near apex; lobes lanceolate-triangular 2.0–2.5 mm wide at the base, 6.0–7.5 mm long, upper part very narrow; fruiting calyx lobes lanceolate, 7–10 × 1.4–2.2 mm. Corolla 4–5-lobed, white; campanulate tube, 3–4 mm long, 2.5–3.0 mm in diameter at the throat, membranous, glabrous on both surfaces; lobes lanceolate, 10–12 × 3.5–4.0 mm, acute or obtuse apex, entire margin, sparsely stellate trichomes like those of the stem on the abaxial surface, glabrous to sparsely few stellate on the adaxial surface, midvein translucent. Stamens 4–5; filament tube < 1 mm long, free portion of the filaments ca. 3.2–3.5 mm long, glabrous; anthers clavate (2D-shaped) with trilongitudinal lobes (3D-shaped), 6–7 mm long, ca. 1.5 mm wide, tapering to apex, anthers not markedly connivent to weakly spreading, yellow, base cordate, apex obtuse, the pores apical, not opening into longitudinal slits with age. Ovary conical, 1.2–1.5 mm diam., glabrous, except for sparsely glandular hairs near apex, biloculate with numerous ovules; style 9–10 mm long, white, glabrous; stigma ca. 0.4 mm wide, green to brown. Fruit a globose berry 1.0–1.2 cm in diameter, green at maturity, glabrous, held downward, with 3–6 fruits per infructescence; completely enclosed by fruiting calyx; fruiting calyx with pale green prickle in living plants; fruiting pedicel 1.2–1.4 cm long, ca. 2 mm diameter, with or without prickles. Seeds reniform, concavo-concave, 3.0–4.0 × 2.5–3.0 mm, brown, 25–40 per fruit.

## 7. Another Specimen Examined

Thailand. Mukdahan Province: Phu Pha Yol National Park, 29 August 2020, W. Chatan 3222 (BK).

## 8. Phenology

The plant produces flowers and fruits during the rainy season, with flowering occurring from June to September and fruiting from August to November.

## 9. Distribution

**Figure 4:**
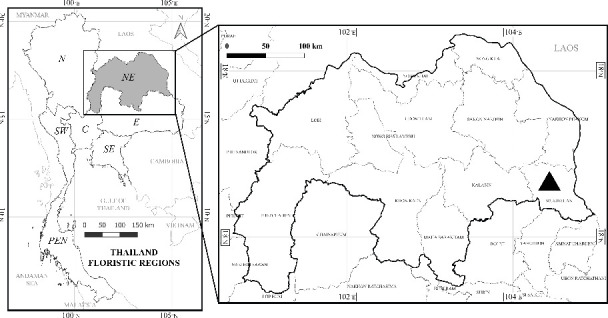
Distribution of *Solanum baumii* Chatan and Promprom, sp. nov. ▲: Phu Pha Yol National Park, Mukdahan Province, Thailand.

The new species is rare and occurs in Thailand, known only from its type locality at Phu Pha Yol National Park, Mukdahan Province, in northeastern Thailand (Figure 4). The newly identified species is endemic to Thailand. In contrast, *S. praetermissum* is distributed in Thailand and also found in South-Central China, Southeast China, the Eastern Himalayas, and Laos. Similarly, *S. barbisetum* Nees occurs in Thailand and extends its range to Assam, Bangladesh, South-Central China, the Eastern Himalayas, Laos, Malaya, Myanmar, and Nepal.

## 10. Ecology and Habitat

The new species thrives along trails in both slightly shaded and fully shaded areas within deciduous dipterocarp and mixed deciduous forests at elevations ranging from 300 to 400 m.

## 11. Etymology

The specific epithet of *S. baumii* honors Dr. Bernard R. Baum from Agriculture and Agri-Food Canada, Eastern Cereal and Oilseed Research Centre, in Ottawa, Ontario, Canada. Dr. Baum, who served as the first author's coadvisor during his PhD, is widely recognized for his extensive contributions to classical and modern plant taxonomy, a field to which he has devoted his entire career.

## 12. Vernacular Name

Ma Khuea Noi.

## 13. Preliminary Conservation Status

Based on data from field trips in northeastern Thailand, only a single population of *S. baumii* has been found at its type locality in Phu Pha Yol National Park, Mukdahan Province. The species has not been observed in any other protected or unprotected areas and occurs along trails that are easily accessible to humans. However, additional data from other regions are still pending and may contribute to future assessments. Therefore, the species should be considered data deficient (DD) according to the IUCN criteria [22].

## 14. Discussion

*Solanum baumii* Chatan and Promprom is similar to *S. praetermissum* Kerr ex Barnett, a plant distributed in East Himalaya to China, Thailand, and Laos [3, 14], and *S. barbisetum* Nees, a plant with the native range from Himalaya to China (Yunnan) and Peninsula Malaysia [3]. The three species share similar morphology, including a shrub habit, straight prickles on the stem, young stems or upper leaf surfaces with stellate pubescence, leaves with variously shallowly lobed or entire margins (not pinnatifid or bipinnatifid), attenuate to truncate leaf bases, simple inflorescences, and usually perfect flowers. Mature berries are glabrous, whitish green or green, usually about 1 cm in diameter, with an accrescent calyx in fruit.

The morphological comparison of the three *Solanum* species reveals distinct differences in various characters. *Solanum baumii* Chatan and Promprom is characterized by stems armed with very few and thin prickles when young, with older stems becoming unarmed and prickles measuring 0.5–1.5 mm long and brownish green in live plants. Its leaves are similarly armed with a few prickles that are 1–3 mm long and brownish green. The inflorescences are 3–4 cm long, bearing 6–18 flowers. The flowers are 4–5-merous, the calyx tube is 5.0–5.5 mm long with brownish-green prickles, and the corolla is always white. The androecium consists of 4–5 stamens that the anther ranges from weakly spreading to not markedly connivent. The ovaries are glabrous except for sparsely glandular hairs near the apex, and the fruits are 1.0–1.2 cm in diameter, green at maturity.

In contrast, *Solanum praetermissum* Kerr ex Barnett has stems with moderate to sparse prickles up to 4 mm long, often purplish black in live plants, and leaves with moderately prickly surfaces. Its inflorescences are 1.5–3.0 cm long, bearing ca. 5–11 flowers, with 5-merous flowers. The calyx tube is 2–3 mm long with purplish-black prickles, and the corolla is white, sometimes pale lilac. The stamens have five anthers that are not markedly connivent. The ovaries are glabrous except for being minutely glandular-puberulent near the apex, and the fruits are 0.8–1.2 cm in diameter, whitish cream at maturity. *Solanum barbisetum* Nees exhibits heavily armed older stems with dense prickles and bristles up to 0.5 cm long, purplish black or yellowish-tan. Its leaves are sparsely prickly with prickles up to 1 cm long. The inflorescences are (1–)3–10 cm long, bearing 10–40 flowers, and the flowers are 5-merous and heterostylous. The calyx tube is 2.5–5.5 mm long with purplish-black prickles, and the corolla is white or violet. The stamens have five anthers that are slightly connivent. The ovaries are entirely glabrous, and the fruits are 1–1.4 cm in diameter, pale greenish white at maturity. The fruiting pedicels vary in length and diameter across the species, and the seeds are flattened-reniform with slight differences in dimensions. The morphological data presented above indicate that the new species is clearly distinct from the two related species. However, its phylogenetic relationship to other *Solanum* species remains unresolved and warrants further investigation to achieve a clearer understanding. Details of morphological differences between *S. baumii* Chatan and Promprom and *S. praetermissum* Kerr ex Barnett are shown in Table 1, and a key to these closely related species is presented below (Table 2).

## Figures and Tables

**Figure 1:**
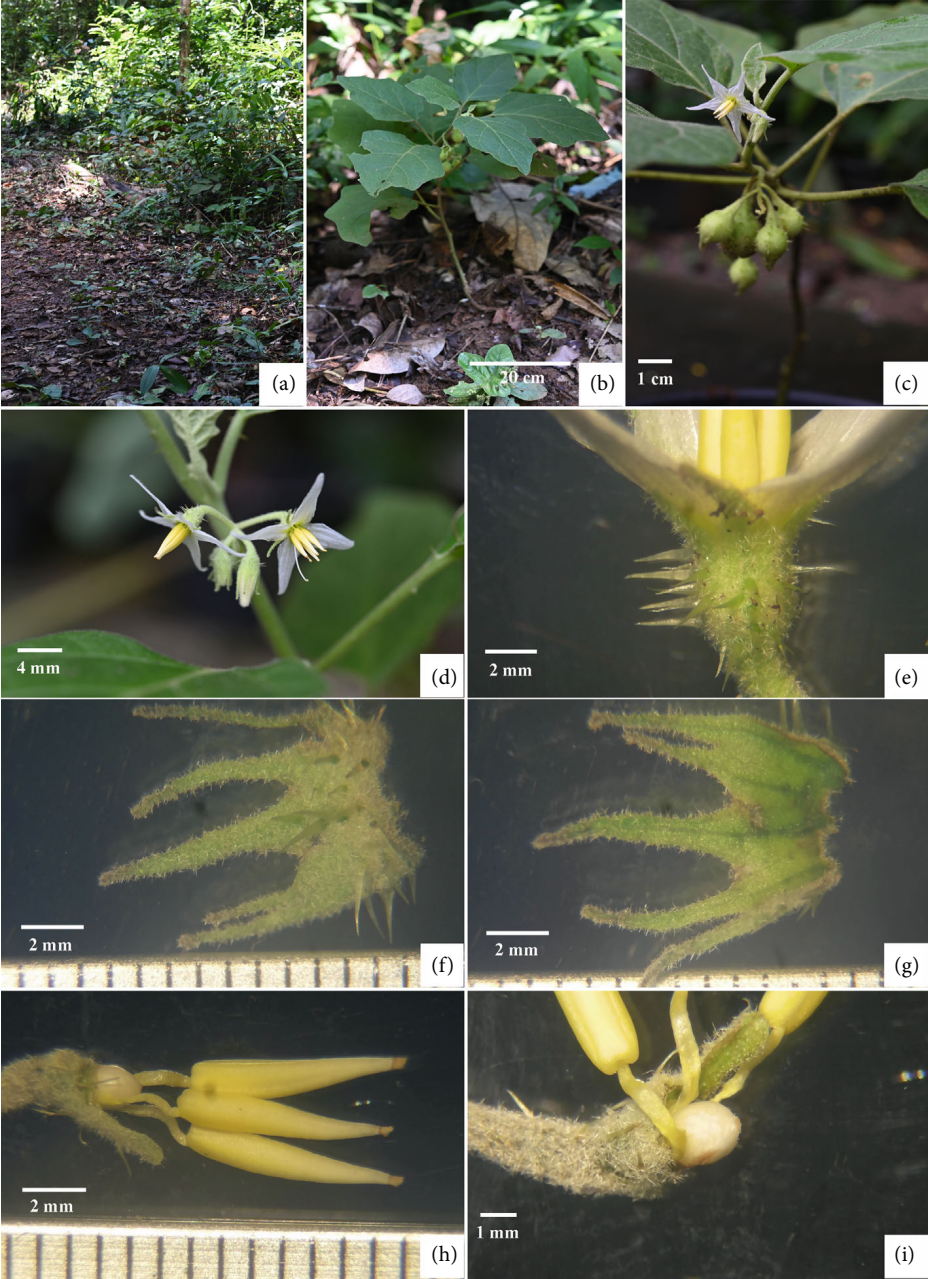
*Solanum baumii* Chatan and Promprom sp. nov. (a) Habitat. (b) Habit. (c) A shoot with inflorescence and fruits. (d) Inflorescence (enlarged view). (e) Calyx in flower. (f) Abaxial side of the calyx. (g) Adaxial side of the calyx. (h) Three stamens and an ovary. (i) Ovary and part of stamens.

**Figure 2:**
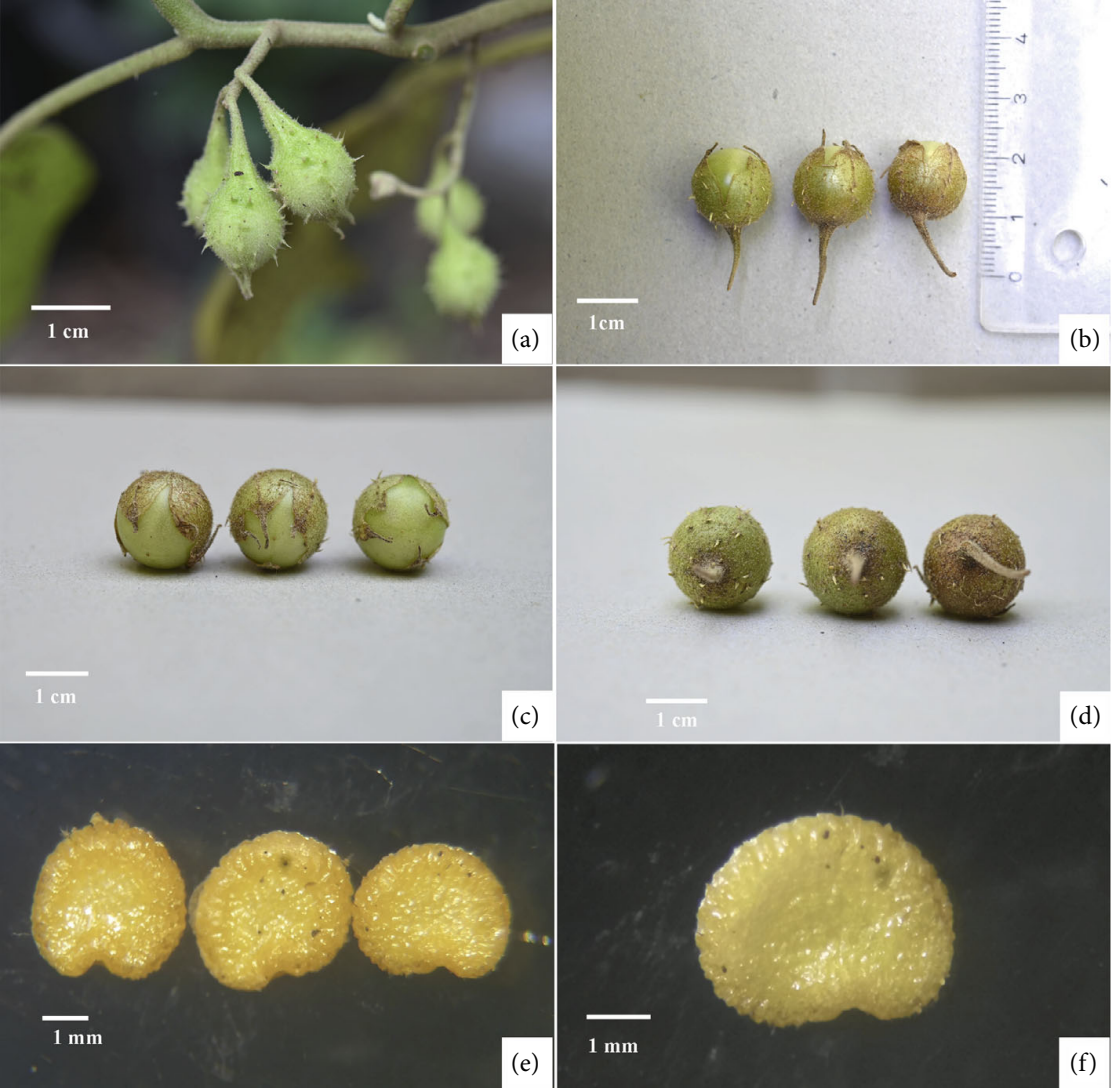
*Solanum baumii* Chatan and Promprom, sp. nov. (a) Young fruits on the plant. (b) Ripe fruit, lateral view. (c) Ripe fruit, top view. (d) Ripe fruit, bottom view. (e) Seeds. (f) Seed, enlarged view (photographs by Wilawan Promprom).

**Figure 3:**
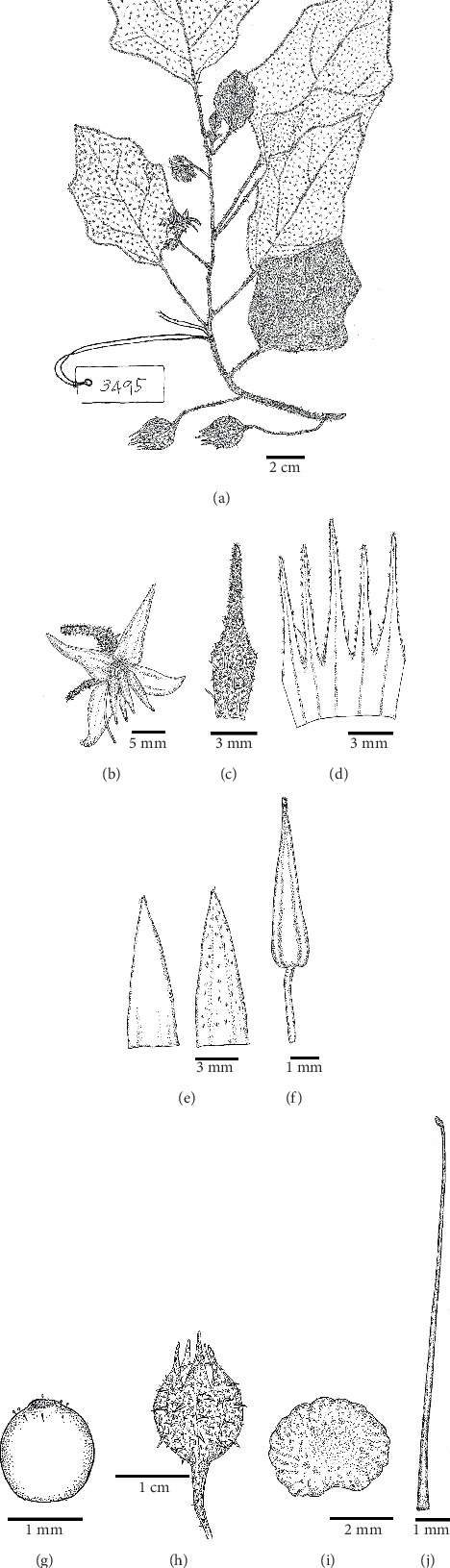
Morphology of *Solanum baumii* Chatan and Promprom, sp. nov. (a) Flowering and fruiting branch. (b) Flower. (c) Calyx (abaxial view). (d) Calyx tube and calyx lobes (adaxial view). (e) Corolla lobes (left: adaxial view; right: abaxial view). (f) Stamen. (g) Pistil. (h) Fruit. (i) Seed. (j) Style and stigma. Illustration by Wannachai Chatan (based on the type specimen).

**Table 1:** Morphological differences among *Solanum baumii* Chatan and Promprom, *Solanum barbisetum* Nees, and *Solanum praetermissum* Kerr ex Barnett.

**Characters**	***S. baumii* Chatan and Promprom**	***S. barbisetum* Nees**	***S. praetermissum* Kerr ex Barnett**
Stems	Armed with very few and thin prickles when young; the older stems unarmed; prickles 0.5–1.5 mm long and brownish green in live plants	Heavily armed with dense, bristly prickles on older stems; prickles of varying sizes up to 0.5 cm long, purplish black or yellowish tan in live plants	Armed with moderate to sparse prickles; prickles up to 4 mm long, pale yellow in live plants; prickles often purplish black in living plants
Leaves	Both surfaces armed with a few prickles; prickles 1–3 mm long and brownish green in living plants	Both surfaces sparsely prickly; the prickles up to 1 cm long and purplish black or yellowish tan in living plants	Both surfaces moderately prickly; the prickles up to 7 mm long and often purplish black in living plants
Inflorescences	3–4 cm long, bearing 6–18 flowers	(1–)3–10 cm long, bearing 10 to 40 flowers	1.5–3.0 cm long, bearing ca. 5 to 11 flowers
Flowers	4–5-merous, apparently all perfect	5-merous, heterostylous and the plants weakly andromonoecious	5-merous, apparently all perfect
Calyx	Tube 5.0–5.5 mm long, adaxial side brownish-green prickles in living plants, cupular with 4–5 lobes; lobes greenish	Tube 2.5–5.5 mm long, adaxial side purplish-black prickles in live plants, deeply cupulate to somewhat urceolate, 5-lobed; lobes greenish	Tube 2–3 mm long, adaxial side purplish-black prickles in living plants, conical with 5-lobed; lobes pale purple
Corolla	4–5-lobed, always white	5-lobed, white or violet (usually adaxially white and abaxially violet, but not always)	5-lobed, white, sometimes pale lilac
Stamens	4–5; filament tube < 1 mm long; free portion of the filaments 3.2–3.5 mm long; anthers not markedly connivent, ranging to weakly spreading	5, filament tube minute; free portion of the filaments minute; the anthers almost sessile and slightly connivent	5; filament tube < 1 mm long; free portion of the filaments 0.5–1 mm long; anthers not markedly connivent
Ovaries	Glabrous, except for sparsely glandular hairs near the apex	Glabrous	Glabrous, except for minutely glandular-puberulent near apex
Styles	9–10 mm long	3–10 mm long	Ca. 6 mm long
Fruits	1.0–1.2 cm in diameter, green at maturity	Fruit 1–1.4 cm in diameter, pale greenish white at maturity	Fruit 0.8–1.2 cm in diameter, whitish cream at maturity
Fruiting pedicels	1.2–1.4 cm in length, approximately 2 mm in diameter	0.7–0.9 cm in length, approximately 1–2 mm in diameter	0.9–1.2 cm in length, approximately 1.0–3.5 mm in diameter
Fruiting calyx prickles	Pale green in living plants	Often purplish black in living plants	Often purplish black in living plants
Seeds	Flattened-reniform, concavo-concave, 3.0–4.0 in length, 2.5–3.0 mm wide	Seeds flattened-reniform, 2.5–5 mm in length, 1.5–3.0 mm in width	Seeds flattened-reniform, 2–3 mm in length, 1.5–2.0 mm in width

**Table 2:** Key to species of the closely related species.

1	Young growth densely covered with prickles; prickles bristly on older stems and branches	*Solanum barbisetum*
1	Young growth sparsely prickly, not bristly	2
2	Prickles on leaves, stems, and calyx, purplish black in living plants; fruits, whitish cream at maturity	*Solanum praetermissum*
2	Prickles on leaves, stem, and calyx, brownish green in living plants; fruits, green at maturity	*Solanum baumii*

## Data Availability

All data generated or analyzed during this study are available within the paper.
